# Deletion of β-Neurexins in Mice Alters the Distribution of Dense-Core Vesicles in Presynapses of Hippocampal and Cerebellar Neurons

**DOI:** 10.3389/fnana.2021.757017

**Published:** 2022-01-31

**Authors:** Shima Ferdos, Johannes Brockhaus, Markus Missler, Astrid Rohlmann

**Affiliations:** Institute of Anatomy and Molecular Neurobiology, Westfälische Wilhelms-University, Münster, Germany

**Keywords:** synapse function, neuropeptides, neuromodulators, electron microscopy, exocytosis, secretion, dense-core vesicles

## Abstract

Communication between neurons through synapses includes the release of neurotransmitter-containing synaptic vesicles (SVs) and of neuromodulator-containing dense-core vesicles (DCVs). Neurexins (Nrxns), a polymorphic family of cell surface molecules encoded by three genes in vertebrates (Nrxn1–3), have been proposed as essential presynaptic organizers and as candidates for cell type-specific or even synapse-specific regulation of synaptic vesicle exocytosis. However, it remains unknown whether Nrxns also regulate DCVs. Here, we report that at least β-neurexins (β-Nrxns), an extracellularly smaller Nrxn variant, are involved in the distribution of presynaptic DCVs. We found that conditional deletion of all three β-Nrxn isoforms in mice by lentivirus-mediated Cre recombinase expression in primary hippocampal neurons reduces the number of ultrastructurally identified DCVs in presynaptic boutons. Consistently, colabeling against marker proteins revealed a diminished population of chromogranin A- (ChrgA-) positive DCVs in synapses and axons of β-Nrxn-deficient neurons. Moreover, we validated the impaired DCV distribution in cerebellar brain tissue from constitutive β-Nrxn knockout (β-TKO) mice, where DCVs are normally abundant and β-Nrxn isoforms are prominently expressed. Finally, we observed that the ultrastructure and marker proteins of the Golgi apparatus, responsible for packaging neuropeptides into DCVs, seem unchanged. In conclusion, based on the validation from the two deletion strategies in conditional and constitutive KO mice, two neuronal populations from the hippocampus and cerebellum, and two experimental protocols in cultured neurons and in the brain tissue, this study presented morphological evidence that the number of DCVs at synapses is altered in the absence of β-Nrxns. Our results therefore point to an unexpected contribution of β-Nrxns to the organization of neuropeptide and neuromodulator function, in addition to their more established role in synaptic vesicle release.

## Introduction

Communication between neurons *via* the release of neurotransmitters and neuromodulators is a key process for normal brain function. These signaling molecules are secreted by two specialized pathways, which usually occur on different time scales: the fast exocytosis of neurotransmitter-containing synaptic vesicles (SVs) is triggered within milliseconds by action potential-induced Ca^2+^ influx into presynaptic boutons (Catterall, 2000; Sudhof, 2012) whereas the slower, protracted release of neuropeptide-containing dense-core vesicles (DCVs) usually requires repetitive stimulations (Hartmann et al., 2001; Gartner and Staiger, 2002) and may involve the activation of additional target molecules such as protein kinase C (Sieburth et al., 2007). The neuropeptide-containing DCVs sustain important functions in synapse formation and plasticity (McAllister et al., 1999; Poo, 2001; van den Pol, 2012). Consequently, impaired DCV signaling has been linked to neuropsychiatric diseases such as autism, schizophrenia, and mood disorders (Meyer-Lindenberg et al., 2011; Kormos and Gaszner, 2013). Recent studies of DCV release from mouse hippocampal neurons have demonstrated that SVs and DCVs both depend on molecules of the neuronal release machinery and its regulators, including synaptobrevin, SNAP-25, RIM, Munc13, and Munc18 (van de Bospoort et al., 2012; Arora et al., 2017; Moro et al., 2021; Puntman et al., 2021). However, there are also important differences between SV and DCV pathways, for example, the continuous *de novo* generation of DCVs from the Golgi apparatus (Wong et al., 2012; Bharat et al., 2017) or the more widely localized fusion sites also outside synapses (Persoon et al., 2018; Moro et al., 2021). The question arises if regulator molecules exist that might play a role in both types of regulated exocytosis from neurons.

The neurexin family of cell surface molecules was proposed as essential synaptic organizers and as candidates for cell type-specific or even synapse-specific regulation (Reissner et al., 2013; Sudhof, 2017; Gomez et al., 2021). Neurexins (Nrxns) are polymorphic molecules encoded by the three genes in vertebrates (*Nrxns1-3*). Each gene contains independent promoters that drive the transcription of structurally larger αNrxns and smaller β-neurexins (β-Nrxns), and more variants arise from up to six conserved alternative splice sites (Schreiner et al., 2014; Treutlein et al., 2014). Nrxns not only occur in multifarious variants but also exhibit a differential distribution throughout brain regions and neuronal subpopulations (Ullrich et al., 1995; Schreiner et al., 2014; Fuccillo et al., 2015; Uchigashima et al., 2019). Interestingly, functional differences exist between the different Nrxns that correspond to specific splice variants, for example, splice site #4, which regulates binding to neuroligins or cerebellins (Reissner et al., 2008, 2013; Sudhof, 2017; Gomez et al., 2021). Extracellularly, αNrxn proteins mostly comprise six Laminin A, Neurexin, Sex hormone-binding protein (LNS) domains with interspersed epidermal growth factor- (EGF-) like repeats. Shorter β-Nrxns differ by expressing a β-specific, 37 residue-long domain before splicing into the last (sixth) LNS domain of the respective gene (Reissner et al., 2013; Sudhof, 2017). As LNS6 and subsequent sequences are identical in α- and β-Nrxns, they share properties such as motifs required for intracellular trafficking (Fairless et al., 2008; Neupert et al., 2015; Lin et al., 2019; Ribeiro et al., 2019), a heparan sulfate glycan moiety (Zhang et al., 2018), and physiological ectodomain cleavage (Trotter et al., 2019; Klatt et al., 2021). In addition, α- and β-Nrxn variants have numerous binding partners such as neuroligins (Ichtchenko et al., 1995; Boucard et al., 2005; Reissner et al., 2008), leucine-rich repeat transmembrane neuronal (LRRTM) proteins (de Wit et al., 2009; Ko et al., 2009; Siddiqui et al., 2010), α-dystroglycan (Sugita et al., 2001; Reissner et al., 2014), latrophilins (Boucard et al., 2012), and cerebellins (Uemura et al., 2010; Matsuda and Yuzaki, 2011) that all interact at the LNS6/single LNS domain, albeit with different preferences or affinities. Together, the highest degree of polymorphism, differential distribution, and binding activities of Nrxns have led to the hypothesis of Nrxns as molecular codes that shape the functional properties of synapses.

In spite of similarities, α- and β-Nrxns differ when their function is probed in deletion mouse models. Removal of a single Nrxn-1α or Nrxn-2α reduces neurotransmission (Etherton et al., 2009; Born et al., 2015) and impairs social behaviors (Etherton et al., 2009; Grayton et al., 2013; Dachtler et al., 2014, 2015; Born et al., 2015). Moreover, the combined knockout (KO) of all three α-Nrxn genes was lethal and was mechanistically traced back to an impairment of Ca^2+^-dependent release of SVs from excitatory and inhibitory synapses (Missler et al., 2003; Kattenstroth et al., 2004; Zhang et al., 2005; Brockhaus et al., 2018). Selective deletion of all extracellularly shorter β-Nrxns, in turn, does not significantly impair survival and displays a milder phenotype of reduced excitatory release (Anderson et al., 2015). While this study proposed an involvement of the endocannabinoid signaling pathway in the phenotype, we recently provided evidence that the β-Nrxn proteins are also involved in the regulation of Ca^2+^ transients and glutamate release, albeit to a lesser degree compared to α-Nrxn proteins (Klatt et al., 2021). We found that endogenous β-Nrxns are present in excitatory and inhibitory synapses but surprisingly occupy only 40% of terminals. Instead, a large population of highly mobile β-Nrxn molecules is dynamically regulated by activity (Klatt et al., 2021). As the release of DCVs occurs mostly in the axonal compartment where it depends less on specialized active zones than SVs (Persoon et al., 2018; Moro et al., 2021), we hypothesized that β-Nrxns may have a hitherto unrecognized, regulatory role in DCV function.

Here, we have combined electron microscopy, immunocytochemistry, and conditional and constitutive KO mice to reveal that the normal distribution of chromogranin A- (ChrgA-) positive DCVs in cultured neurons and brain tissue depends on the presence of β-Nrxn proteins. Our findings indicate that β-Nrxns, in addition to regulating Ca^2+^-depending release of SVs and to providing synaptogenic properties, may serve a role in DCV function.

## Materials and Methods

### Animals

For the deletion of β-Nrxns, we used a previously described triple β-Nrxn floxed mouse strain (βKI; available from JAX Labs as B6;129-Nx1^TM 2*Sud*^ Nx2^TM 2*Sud*^ Nx3^TM 2*Sud*^/J, RRID:IMSR_JAX:008416) (Anderson et al., 2015). In βKI mice, the 5′-exons specific for β-Nrxns are flanked by two loxP sites, which can be recognized and subsequently excised by Cre recombinase introduced, for example, by lentivirus to create a conditional knockout (cKO). In addition, a constitutive triple β-Nrxn knockout (β-TKO) was generated by crossing B6;129-Nx1^TM 2*Sud*^ Nx2^TM 2*Sud*^ Nx3^TM 2*Sud*^/J with transgenic mice B6.FVB-Tg (Ella-Cre) C5379Lmgd/J (RRID:IMSR_JAX:003724) expressing Cre recombinase. The βTKO mice were subsequently bred to C57BL/6J (RRID:IMSR_JAX:000664) for several generations to remove Ella-Cre transgene. All animal experiments were carried out at the University of Münster according to government regulations for animal welfare and approved by the Landesamt für Natur, Umwelt und Verbraucherschutz (LANUV, NRW, Germany), license numbers 84-02.05.20.11.209 and 84-02.04.2015.A423.

### Lentivirus Production and Infection

The lentivirus system expressing active or inactive Cre recombinase fused to enhanced green fluorescent protein (EGFP) used here was derived from the laboratory of Tom Südhof and was used in primary hippocampal or cortical cultures to effectively delete, for example, Mint proteins (Ho et al., 2006), RIM proteins (Kaeser et al., 2011), splice inserts of Nrxns (Aoto et al., 2013, 2015), or entire neurexin genes (Anderson et al., 2015; Trotter et al., 2019; Klatt et al., 2021), as shown by immunoblots for complete deletion of the respective genes. The production of lentiviruses and infection of neuronal cultures with lentiviruses in our lab have been previously described (Klatt et al., 2021). Briefly, the helper plasmids pRSV-REV (RRID:Addgene_12253), pMDLg/gRRE (RRID:Addgene_12251), and pVSVG (RRID:Addgene_8454) were co-transfected with lentiviral expression vectors [FSW-NLS-GFP-Cre or FSW-NLS-GFP-Cre*^mut^* (Y324F)] (de Jong et al., 2018; Klatt et al., 2021) into the human embryonic kidney (HEK) 293T cells using Lipofectamine 2000 (Thermo Fisher Scientific, Waltham, MA, United States, Cat #11668019). About 72 h after transfection, viral supernatant was collected and centrifugated at 500 g for 10 min at 4°C. The supernatant was aliquoted and immediately frozen at -80°C with maximal storage time of 4 weeks. At 5 days *in vitro* (DIV5), 150 μl of lentiviral supernatant (Cre or Cre*^mut^*) was added dropwise to neuronal cultures for 3 days. Neurons infected either with Cre- or Cre*^mut^*-lentiviruses could be detected by nuclear expression of EGFP driven by a synapsin promoter. Based on the GFP-Cre or GFP-Cre*^mut^* autofluorescence and colabeling with MAP2 as a marker of neuronal cell bodies and dendrites (see section “Immunocytochemistry”), we routinely tested virus preparations for the effectiveness of transduction. We normally determined the efficiency between 92 and 99% and excluded preparations below 95% from this study.

### Neuronal Cell Culture

Hippocampal neurons were prepared in HBSS from β-Nrxn floxed mice as described (Brockhaus et al., 2018). Briefly, primary hippocampal neurons derived from timed-pregnant dams at E17.5 were dissociated by 0.25% trypsin and plated onto 18-mm glass coverslips (Menzel-Glaseser) coated with poly-L-lysine (Sigma-Aldrich, St. Louis, MO, United States, Cat #P1524) at the density of 55,000 cells/coverslip in plating medium. After 3–4 h at 37°C neuronal plating medium containing minimum essential medium (MEM), 10% horse serum, 0.6% glucose, and 1 mM sodium pyruvate, coverslips were inverted onto a 70–80% confluent monolayer of mouse astrocytes grown in 12-well plates (Falcon), and incubated in neurobasal medium (plus B27, 0.5 mM glutamine, and 12.5 μM glutamate). After 3 days, media were refreshed with neurobasal medium (GIBCO, ThermoFisher Scientific, Waltham, MA, United States, Cat #21103-049) supplemented with B27, 0.5 mM glutamine, and 5 μM AraC. Cultured neurons were maintained at 37°C in a humidified incubator with an atmosphere of 95% air and 5% CO_2_.

### Immunocytochemistry

Fixation of cultured neurons at DIV 18 was performed with 4% formaldehyde and 4% sucrose in phosphate buffered saline (PBS) (pH 7.4) for 15 min at RT. After several washing steps, fixed cells were incubated in a blocking solution containing 5% next-generation sequencing (NGS) and 0.3% Triton X-100 in PBS for 30 min to block non-specific interactions. Primary or secondary antibodies were diluted in antibody solution (5% NGS in PBS). Coverslips were incubated with primary antibodies for 2.5 h, followed by washing steps. Incubation with secondary antibodies was done for 1 h. Finally, coverslips were mounted using Deko fluorescence mounting medium and visualized by fluorescence microscope with 63× oil immersion objective and maximal resolution. Fluorescent intensity measurement was performed using the ImageJ software (NIH Image, RRID:SCR_003073).

#### Primary Antibodies

Polyclonal rabbit ChrgA (1:500; Synaptic System Cat #259003, RRID:AB_2619972), polyclonal chicken MAP2 (1:500; Abcam Cat #ab5392, RRID:AB_2138153), polyclonal chicken neurofilament H (NFH, 1:3000; Synaptic System Cat #171106, RRID:AB_2721078), monoclonal mouse syntaxin-6 (1:250; BD Bioscience Cat #610635, RRID:AB_397966), monoclonal mouse GM130 (1:500; BD transduction laboratories Cat #610822, RRID:AB_398141), and polyclonal guinea pig Bassoon (1:500; Synaptic System Cat #141004, RRID:AB_2290619) were used.

#### Secondary Antibodies

Goat anti-rabbit conjugated to Cy3 (1:500; Jackson ImmunoResearch Labs, West Grove, PA, United States, Cat #111-165-003, RRID:AB_2338000), goat anti-rabbit conjugated to Alexa fluor 488 (1:500; Thermo Fisher Scientific, Waltham, MA, United States, Cat #A-11034, RRID:AB_2576217), goat anti chicken conjugated to Alexa fluor 647 (1:500; Thermo Fisher Scientific, Waltham, MA, United States, Cat #A21449, RRID:Ab_2535866), goat anti-chicken conjugated to Alexa fluor 488 (1:500; Thermo Fisher Scientific, Waltham, MA, United States, Cat #A11039, RRID:AB_2534096), goat anti-mouse conjugated to Cy3 (1:500, Jackson ImmunoResearch Labs, West Grove, PA, United States, Cat #115-165-003, RRID:AB_2338680), goat anti-guinea pig conjugated to Cy3 (1:500; Jackson ImmunoResearch Labs, West Grove, PA, United States, Cat #106-165-003, RRID:AB_2337398) were used.

### Transmission Electron Microscopy and Data Analysis

Samples from cultured neurons and cerebellar tissue were examined with a transmission electron microscope (Libra 120, Zeiss, Jena, Germany) at 80 kV, and imaged with a 2,048 × 2,048 CCD camera (Tröndle, Moorenweis, Germany). The number of DCVs was counted in axonal areas (225 μm^2^) of cultured hippocampal neurons (*n* = 3 virus infection experiments) and 350 μm^2^/total number of synapses in the cerebellar cortex.

#### Sample Preparation From Neuronal Cell Culture

Cultured hippocampal neurons infected with Cre- or Cre*^mut^*-lentiviruses were fixed at DIV24 for 15 min at RT with 0.1% glutaraldehyde (Serva, Heidelberg, Germany) and 2% paraformaldehyde (Merck, Kenilworth, NJ, United States) in 0.1 M PB. After post-fixation with 1% osmium (OsO_4_) solution for 1 h, neurons were briefly washed with dH_2_O and dehydrated in a graded series of ethanol solutions. Cells were incubated with propylene oxide (Electron Microscopy Science) for 15 min, infiltrated with propylene oxide/epon (1:1) for 30 min, embedded in epon resin (EMS) for 3 h, and polymerized at 60°C for 24 h. Following serial ultrathin sectioning (70 nm) on an ultramicrotome [Electron Microscopy (EM), UC6; Leica, Wetzlar, Germany], sections were mounted on formvar-coated copper grids and stained in uranyl acetate and lead citrate.

#### Sample Preparation From Brain Tissue

Under deep anesthesia, constitutive βTKO mice and βKI controls (10 weeks old) were transcardially perfused with prewarmed 0.1 M PB (pH 7.3), followed by a mixture of 4% PFA and 2.5% GA in 0.1 M PB. Brains were dissected and immersed in the same fixative solutions at 4°C overnight. Cerebella were cut in 300 μm coronal slices (Leica, Wetzlar, Germany, VI1000S) and washed. Cerebellar slices were contrasted with 1% OsO_4_ solution for 2 h and dehydrated in a graded series of ethanol solutions. Following incubation with propylene oxide (Electron Microscopy Science) for 45 min at RT, slices were infiltrated with a mixture of propylene oxide/epon (1:1) for 1 h, embedded in epon resin (EMS) for 1 h, and polymerized at 60°C for 24 h. Ultrathin sections (70 nm) were prepared on an ultramicrotome (EM, UC6; Leica, Wetzlar, Germany).

#### Electron Microscopy Image Analysis

For area size of boutons of primary hippocampal neurons and parallel fiber terminals in the cerebellum, presynaptic areas containing SVs were circled using the TEM morphometry software (Tröndle, Moorenweis, Germany) and areas were calculated. In the same synapses, the number of SVs was counted and active zone length was determined. The sample area analyzed was aimed at about 1.000 μm^2^ per animal and genotype, amounting to 3.375 μm^2^ of hippocampal neurons per genotype and 3.123 μm^2^ cerebellar tissue per genotype (cerebellum). This sampling procedure was repeated three times in independent cultures or three animals per genotype.

### Light-Microscopic Imaging

#### Fluorescent Microscopy and Data Analysis

Immunofluorescent images were acquired on a VisiGRID fluorescence microscope equipped with oil immersion objective (x63, NA 1.4), SPOT pursuit (Sony Xplorer-XS) camera, and the VisiView software (Visitron Systems, Puchheim, Germany). To compare the relative ChrgA fluorescent intensity between Cre- and Cre*^mut^*-infected neurons, 5 axonal profiles per Cre virus infection experiment (*n* = 3 experiments) were analyzed. The fluorescent intensity of ChrgA was measured over approximately 20 μm axon length using the ImageJ software. The measured signal intensity was then normalized to the mean value of ChrgA immunosignal of control neurons (Cre*^mut^*).

#### Bright-Field Microscopy and Data Analysis

For Nissl staining, 0.1% cresyl violet acetate was used on 30-μm-thick cryosections (Leica, Wetzlar, Germany, CM 3050S) of entire brains from βTKO and βKI mice (12 weeks old) that were perfusion-fixed in 4% paraformaldehyde, post-fixed overnight in the same fixative, cryoprotected in 30% sucrose, and embedded in OCT compound (Thermo Fisher Scientific, Waltham, MA, United States).

Cell survival of cerebellar granule cells (CGCs) was examined by counting the number of cells per 1 mm^2^ area on cerebellar semithin sections stained with toluidine blue using light microscopy equipped with a MikroCam PRO HDMI camera.

### Electrophysiological Recordings

Coverslips with cultured hippocampal neurons (DIV 17–18) were placed in the recording chamber of an inverted microscope (Observer.A1, Zeiss, Jena, Germany) with a bath solution (32°C) containing (in mM): 145 NaCl, 3 KCl, 2 CaCl_2_, 1 MgCl_2_, 11 glucose, 10 HEPES, and pH 7.4 adjusted with NaOH. For recordings of pharmacologically isolated miniature excitatory postsynaptic currents (mEPSCs), 10 μM bicuculline and 1 μM tetrodotoxin were added. Whole-cell patch clamp measurements from neurons with a pyramidal cell-like shape were done with a 2.5–3 MΩ pipette containing (in mM): 140 K-gluconate, 1.5 MgCl_2_, 1 CaCl_2_, 10 HEPES, 4 Na-ATP, 0.5 Na-GTP, 10 EGTA, and pH 7.3 with KOH.

Continuous voltage clamp recordings at −70 mV holding potential with 10 kHz sampling rate, filtered at 3.3 kHz, were obtained with an EPC10 amplifier and the Pulse software (HEKA, Reutlingen, Germany). About 100 consecutive mEPSCs per neuron were identified and evaluated for inter-event interval (IEI), amplitude, rise-time (10–90%), and half-width with TaroTools in IgorPro (Wavemetrics). TaroTools is a set of procedures for IgorPro to analyze neurophysiological data, and was written by Taro Ishikawa, Jikei University School of Medicine, Tokyo, Japan. The tool integrates into IgorPro as an add-in and can be downloaded upon request as four separate files from https://sites.google.com/site/tarotoolsregister/registration.

### Statistical Analysis

Statistical analysis of each experiment was performed with the GraphPad Prism software (GraphPad Prism, RRID:SCR_002798). Data are shown as mean ± SEM. To assess the statistical significance between the two groups, we used a two-tailed unpaired Student’s *t*-test. Significance differences are indicated in detail in the corresponding figure legends.

## Results

In mouse hippocampal neurons, DCVs are predominantly released from axonal fusion sites (Persoon et al., 2018; Moro et al., 2021). We recently observed that endogenous β-Nrxns, cell adhesion molecules with important roles in the release of SV, dynamically diffuse at the axonal surface between synaptic and extrasynaptic locations of these neurons (Klatt et al., 2021). To address the question if β-Nrxns also affect DCV distribution, we studied the neurons that lack β-Nrxns and mostly focused on DCVs in presynaptic boutons.

### Distribution of Dense-Core Vesicles Is Altered in β-Neurexin-Deficient Cultured Hippocampal Neurons

To delete β-Nrxns in primary hippocampal neurons, we transduced cultures from triple βNrxn-floxed mice (βKI) at DIV 5 with lentivirus expressing active Cre recombinase (Cre). Conditional deletion of floxed β-specific exons by Cre-expressing lentivirus allows removal of β-Nrxns in comparison to inactive Cre recombinase (Cre*^mut^*) or β-KI neurons as controls. We adopted and characterized an extensively used lentivirus system (see section “Lentivirus Production and Infection”) previously and demonstrated the effectiveness of recombination events and complete deletion of β-Nrxns by immunoblots (Klatt et al., 2021). Consequently, here the distribution of DCVs was studied at DIV18, using the same culture conditions and procedures as in our earlier report (Klatt et al., 2021).

**FIGURE 1:**
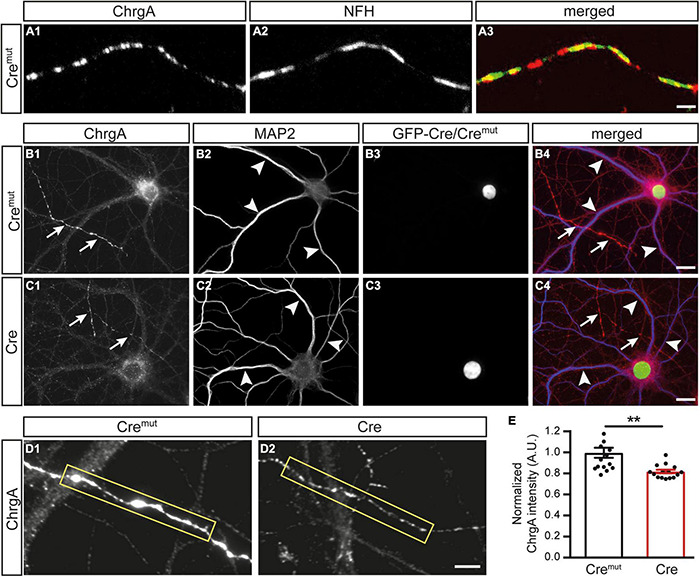
Diminished chromogranin A (ChrgA) levels in β-neurexins- (β-Nrxns-) deficient hippocampal neurons. **(A)** Primary hippocampal neurons from floxed β-Nrxn knock-in (β-KI) mice were transduced with lentivirus expressing inactive Cre recombinase (Cre*^mut^*) and immunostained at DIV18 with anti-ChrgA **(A_1_)** and anti-neurofilament H (NFH, **A_2_**). Merged image **(A_3_)** shows the presence of the dense-core vesicle (DCV) marker ChrgA in axons. Scale bar: 3 μm. **(B,C)** Cultured neurons as in panel **(A)** but colabeled for ChrgA **(B_1_,C_1_)** and the dendritic marker MAP2 **(B_2_,C_2_)**. Successful transduction of neurons with lentivirus is demonstrated by nuclear expression of inactive Cre*^mut^* or active Cre recombinase (Cre) fused to GFP **(B_3_,C_3_)**. Merged images **(B_4_,C_4_)** show the exclusion of ChrgA (arrows) from MAP-positive dendrites (arrowheads) independent of the presence of β-Nrxn. Scale bars: 20 μm. **(D)** Representative images of ChrgA-positive axons of Cre*^mut^* control **(D_1_)** and Cre transduced conditional knockout (cKO) **(D_2_)** neurons from experiments as in panel **(A)** that were used for measurements of fluorescence intensity (yellow boxes). Note that the fluorescence intensity appears to be overall reduced in β-Nrxn-deficient axons with no change in the labeling pattern and without apparent ectopic accumulation. Scale bar: 4 μm. **(E)** Histogram comparing the ChrgA fluorescence intensity over axons from Cre*^mut^* control (black bars) and β-Nrxn-deficient Cre neurons (red bars) as shown in panel **(D)**. Intensities (AU = arbitrary units) were normalized to control values. Data are shown as mean ± SEM, measurements are based on *n* = 15 axonal regions from the three independent cultures per genotype; significance difference indicated as **p < 0.01, two-tailed unpaired *t*-test.

We first monitored the normal distribution of DCVs in control neurons (Cre*^mut^*) by immunolabeling ChrgA which is a common matrix protein of DCVs (Bharat et al., 2017; Dominguez et al., 2018). Colabeling of ChrgA with antibodies against NFH as a specific intermediate filament component of mature axons reliably revealed ChrgA-positive clusters in the identified axons (Figures 1A_**1**_,A_**2**_), resulting in a partly overlapping pattern (Figure 1A_**3**_). Conversely, when ChrgA (arrows, Figure 1B_**1**_) was co-stained in control neurons for the dendritic marker MAP2 (arrowheads, Figure 1B_**1**_), it did not overlap with ChrgA labeling. We also observed that only a subset of axons was ChrgA-positive, which is in accordance with a study showing that DCVs occur in only 18% of axonal boutons in hippocampal slices (Sorra et al., 2006). Successful infection of the labeled neurons by lentivirus with Cre*^mut^* recombinase could be controlled by a green-fluorescent nucleus due to the expression of GFP as a fusion protein with Cre*^mut^* (Figures 1B_**3**_,B_**4**_). We then performed the same set of labelings with cultures infected by active Cre to analyze β-Nrxn-deficient (cKO) neurons (Figures 1C_**1**_–C_**4**_). We compared the effectiveness of GFP-Cre and GFP-Cre*^mut^* expression rates in cultured neurons colabeled by MAP2 antibodies as shown in Figures 1B_**2**_,B_**3**_,C_**2**_,C_**3**_, and determined indistinguishable coexpression rates (mean ± SEM for Cre*^mut^* control: 96.5% ± 0.85, Cre cKO: 96.2% ± 0.78, *n* = 3 cultures per genotype; *p* = 0.9193, two-tailed unpaired *t*-test). Importantly and similar to control, we found that ChrgA labeling was not overlapping with MAP2-positive dendrites in cKO neurons (arrows and arrowheads, Figures 1C_**1**_,C_**2**_), indicating that the deletion of β-Nrxns does not lead to the re-distribution of ChrgA-positive DCVs. Robust nuclear labeling of GFP-Cre recombinase (Figures 1C_**3**_,C_**4**_) again confirmed that ChrgA/MAP2-positive neurons were successfully infected by Cre lentivirus. However, the identified axons of cKO neurons contained less abundant ChrgA staining if compared to controls (Figures 1D_**1**_,D_**2**_). To validate this hypothesis, we measured the intensity of ChrgA fluorescence within a defined axonal window (yellow box, Figures 1D_**1**_,D_**2**_). The results revealed a 20% reduction of ChrgA fluorescence intensity in axons of β-Nrxn cKO neurons compared to control neurons [Figure 1E; data normalized to Cre*^mut^* control: 1.0 ± 0.05 arbitrary units (AU), Cre cKO: 0.82 ± 0.02, *p* = 0.0014]. These data show that the presence of β-Nrxns is required for normal levels of ChrgA in axons of hippocampal neurons, a surprising finding because β-Nrxns have not been linked to DCVs earlier.

**FIGURE 2:**
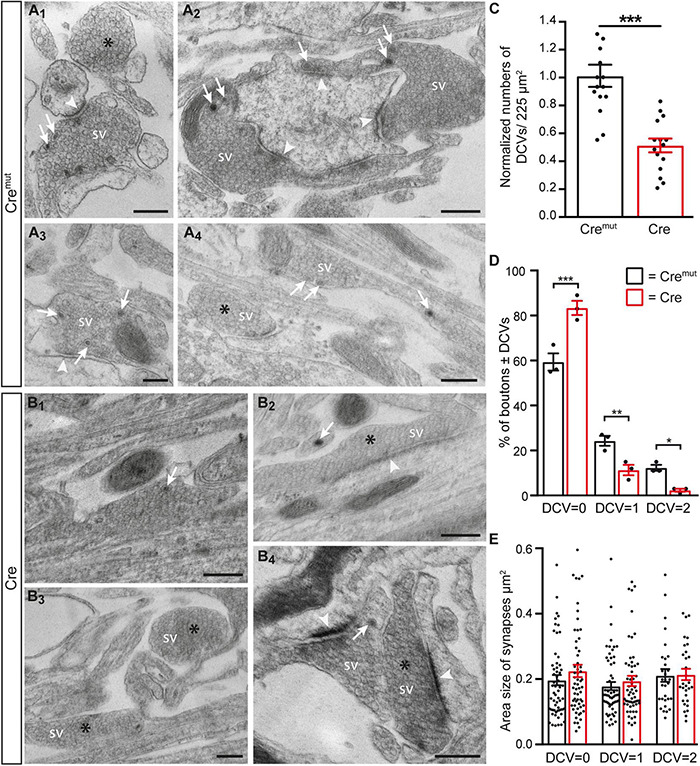
Reduced number of DCVs in presynaptic terminals of β-Nrxn-deficient hippocampal neurons. **(A)** Representative transmission electron microscopic images of synaptic boutons from primary hippocampal neurons of floxed β-KI mice at DIV18 that were transduced with lentivirus expressing inactive Cre recombinase (Cre*^mut^*). Presynapses of these cultured control neurons often contain 1 or more DCVs (arrows, **A_1_–A_4_**). Boutons without DCV are marked by asterisks **(A_1_,A_4_)**. SV, synaptic vesicle clusters; arrowheads, postsynaptic densities. Scale bar: 250 nm. **(B)** Ultrastructure of synaptic boutons as in panel **(A)** but from β-Nrxn-deficient (Cre) neurons revealing fewer presynapses with DCVs (arrows, **B_1_,B_2_,B_4_**) and more without DCVs (asterisks, **B_2_–B_4_**). Scale bar: 250 nm. **(C)** Histogram showing the area density of DCVs in synaptic boutons of Cre*^mut^* control (black bars) and β-Nrxn-deficient Cre (red bars) neurons as shown in panels **(A,B)**. Numbers of DCVs were normalized to control values (for actual values, see section “Results”). Data are shown as mean ± SEM; dots indicate individual data points, measurements are based on *n* = 15 areas from three independent cultures per genotype; significance difference indicated as ***p < 0.0001, two-tailed unpaired *t*-test. **(D)** Histogram displaying the percentage of presynaptic boutons without DCV = 0, a DCV = 1, or DCV = 2. on random cross-sections of Cre*^mut^* control (black bars) and β-Nrxn-deficient Cre (red bars) cultures as analyzed in panel **(C)**. Note the increase of synaptic profiles without DCVs upon the deletion of β-Nrxn. Data are shown as mean ± SEM; dots indicate individual data points. The relative distribution is based on *n* = 162 (DCV = 0), *n* = 64 (DCV = 1), and *n* = 32 (DCV = 2) boutons of Cre*^mut^* neurons, and on *n* = 235 (DCV = 0), *n* = 33 (DCV = 1), and *n* = 7 (DCV = 2) boutons of Cre neurons from the three independent cultures per genotype; significance difference indicated as ***p < 0.0001, **p < 0.01, or *p < 0.05, one-way ANOVA with Holm–Sidak’s multiple comparison. **(E)** Similar histogram to panel **(D)** showing the average area size of boutons without or with DCVs from Cre*^mut^* control (black bars) and β-Nrxn-deficient Cre (red bars) neurons. Data are shown as mean ± SEM. Measurements are based on *n* = 60 (DCV = 0 and DCV = 1) or *n* = 30 (DCV = 2) boutons from the three independent cultures per genotype; significance difference indicated as n.s. = non-significant, two-tailed unpaired *t*-test.

The reduction of ChrgA fluorescence in axons of Cre-infected cultures (Figure 1) raised the question if this was caused by diminished incorporation of the matrix protein into DCVs or a *bona fide* reduction of the number of DCVs in β-Nrxn-deficient neurons. To distinguish between the possibilities, we analyzed the distribution of ultrastructurally identified DCVs in the same hippocampal cultures (Figure 2). While axons of these cultured primary hippocampal neurons are extremely thin, they regularly form presynaptic terminals as *en passant* boutons that line up at close distances of about 5 μm (Staras and Branco, 2010; Staras et al., 2010). We, therefore, focused our electron microscopic analysis on the presynaptic boutons in which typical DCVs could easily be recognized that had a diameter of about 80 nm (Cre*^mut^* control: 83.9 ± 2.26 nm, Cre cKO: 79.9 ± 1.9, *n* = 90 DCVs from three cultures per genotype; n.s., two-tailed unpaired *t*-test), in line with earlier studies (Sorra et al., 2006). In control neurons (Cre*^mut^*), DCVs were frequently located in typical boutons with presynaptic vesicle clusters (arrows, Figures 2A_**1**_–A_**4**_), clearly discernable synaptic cleft and postsynaptic density. DCV-containing boutons were interspersed with those without any visible DCV (asterisks, Figures 2 A_**1**_,A_**4**_). While the general distribution pattern was similar in Cre-infected cKO neurons, the number of DCVs in presynaptic boutons of β-Nrxn cKO seemed lower, very rarely exceeding a single DCV per synapse (arrows, Figures 2B_**1**_,B_**2**_,B_**4**_). Moreover, we also encountered more boutons that were devoid of any DCV in cKO cultures (asterisks, Figures 2B_**2**_–B_**4**_). Quantification of the area density of DCVs confirmed this observation because there was a 50% reduction in cKO (Cre) neurons compared to Cre*^mut^* controls (Figure 2C; Cre*^mut^*: 29.1 ± 2.3 DCVs/225 μm^2^, Cre: 14.7 ± 1.4, *p* < 0.0001). This reduction of the area density of DCVs seemed to be based on a shift from boutons that contain one or two DCVs to synapses with no DCV in β-Nrxn-deficient neurons. In support, counting the respective numbers of presynaptic boutons containing 0, 1, or 2 DCVs revealed a 40% higher number of terminals with zero DCVs in β-Nrxn-deficient neurons (Figure 2D; Cre*^mut^*: 59.3 ± 3.84, Cre: 83.3 ± 3.18, *p* < 0.0001) whereas the number of those containing 1 or 2 DCVs was clearly diminished by about 50 or 80%, respectively (Figure 2D; for DCV = 1 Cre*^mut^*: 24.3 ± 2.19, Cre: 11.3 ± 2.33, *p* = 0.0062; for DCV = 2 Cre*^mut^*: 12.3 ± 1.33, Cre: 2.3 ± 0.67, *p* = 0.015). Interestingly, the shift from boutons that contain DCVs to terminals with no DCV was not reflected by changes in the area size of these boutons (Figure 2E). Area size was indistinguishable in β-Nrxn-deficient Cre and Cre*^mut^* control neurons for DCV = 0 (Figure 2E; Cre*^mut^*: 0.2 ± 0.02, Cre: 0.23 ± 0.02, *p* = 0.3141), for DCV = 1 (Figure 2E; Cre*^mut^*: 0.18 ± 0.01, Cre = 0.2 ± 0.02, *p* = 0.7094), and for DCV = 2 (Figure 2E; Cre*^mut^*: 0.21 ± 0.02, Cre = 0.22 ± 0.02, *p* = 0.8229). Thus, our electron microscopic results demonstrated that the diminished ChrgA fluorescence described in Figure 1 is due to an actual reduction of the number of presynaptic DCVs in the absence of β-Nrxns, leading to a higher number of boutons without any DCV. Our results could imply that the capacity of releasing neuromodulators is strictly controlled in hippocampal neurons, and that shifting of subpopulations of boutons from 1 or 2 DCVs to 0 DCV or *vice versa* might be used to modulate the function of the neuronal network.

**FIGURE 3:**
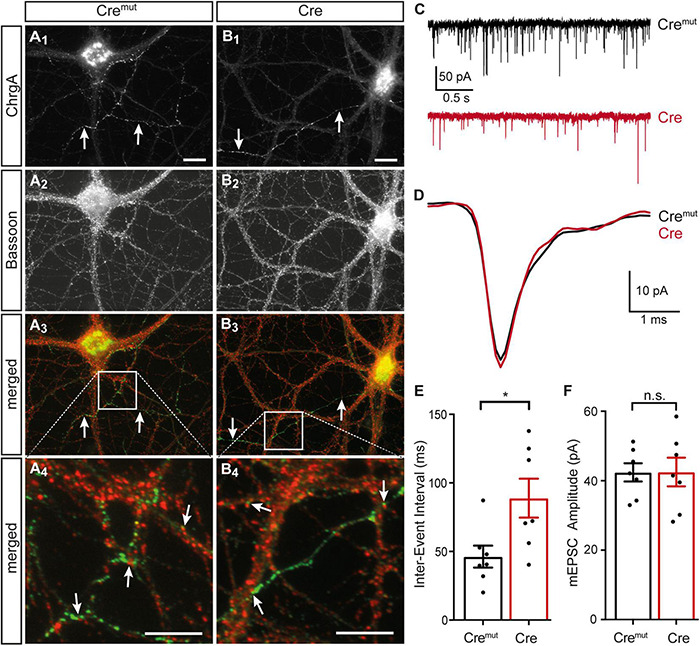
Normal ChrgA-to-Bassoon alignment in synapses of β-Nrxn-deficient neurons. **(A,B)** Primary hippocampal neurons from floxed β-KI mice were transduced with lentivirus expressing inactive Cre*^mut^* **(A_1_–A_4_)** or active Cre recombinase **(B_1_–B_4_)** and immunostained at DIV18 with anti-ChrgA (arrows, **A_1_,B_1_**) and anti-Bassoon (**A_2_,B_2_**). Merged images (**A_3_,B_3_**) show the more restricted expression of the DCV marker ChrgA (arrows, green label) in comparison to the ubiquitous punctate distribution of the presynaptic marker Bassoon (red label). Note the juxtaposed localization of these molecules in high magnification images (arrows, **A_4_,B_4_**). Scale bars in panels **(A_1_,B_1_)** for panels **(A_1_–A_3_,B_1_–B_3_)**: 20 μm; scale bars in panels **(A_4_,B_4_)**: 8 μm. **(C)** Representative traces of whole-cell patch clamp recordings of pharmacologically isolated miniature excitatory postsynaptic currents (mEPSCs) show a visibly reduced frequency of mini events in β-Nrxn-deficient (Cre, red) neurons compared to controls (Cre*^mut^*, black). **(D)** Averaged individual mEPSC traces from more than 100 consecutive events reveal similar amplitudes and kinetics in β-Nrxn-deficient (Cre, red trace) neurons compared to controls (Cre*^mut^*, black trace). **(E,F)** The reduced mEPSC frequency in Cre neurons as shown in panel **(C)** is reflected by longer inter-event intervals (IEIs) **(E)**, whereas the mEPSC amplitude does not differ between genotypes **(F)**. Data are shown as mean ± SEM, dots indicate individual data points, measurements are based on *n* = 7 Cre*^mut^* control neurons with 647 mEPSC events (black bar) and *n* = 7 β-Nrxn-deficient Cre neurons with 575 mEPSCs (red bar) from the 3 independent cultures per genotype; significance difference indicated as ***p < 0.0001 or n.s. = non-significant, two-tailed unpaired *t*-test.

The presence of DCVs within or near presynaptic boutons is an important aspect because they represent the specific release sites for DCVs in hippocampal neurons where they are mostly found at the periphery of the synaptic vesicle clusters (Persoon et al., 2018). As the deletion of β-Nrxns has an effect on synaptic vesicle release (Anderson et al., 2015; Klatt et al., 2021), we tested if the alignment of ChrgA-positive DCVs with the active zone component Bassoon was altered in cKO neurons. Colabeling of ChrgA and Bassoon revealed prominent presence of ChrgA only in a subpopulation of axons (arrows, Figures 3A_**1**_,B_**1**_) as also seen above in ChrgA labelings (Figures 1B_**1**_,C_**1**_). In contrast, Bassoon immunostaining showed a ubiquitous punctate pattern (Figures 3A_**2**_,B_**2**_), as expected from this canonical presynaptic marker protein (Gundelfinger et al., 2015; Biederer et al., 2017). Importantly, the general patterns of ChrgA and Bassoon distribution did not differ between controls (Cre*^mut^*) and β-Nrxn-deficient (Cre) neurons. Furthermore, the overlay of ChrgA and Bassoon immunofluorescence images at lower and higher resolution (merged, Figures 3A_**3**_,A_**4**_,B_**3**_,B_**4**_) revealed that DCVs and active zone components are mostly juxtaposed with very little or no overlap (arrows, Figures 3A_**4**_,B_**4**_). These findings are in accordance with the earlier data that reported an average distance of 0.34 μm of DCVs from the active zone (Persoon et al., 2018). In addition, extrasynaptic staining of ChrgA outside Bassoon-positive boutons indicated that DCVs are trafficking along an axon (Figures 3A_**3**_,A_**4**_,B_**3**_,B_**4**_) both in controls (Cre*^mut^*) and β-Nrxn-deficient (Cre) neurons. They are supposed to be transported bidirectionally and captured sporadically in en passent synapses (Wong et al., 2012).

To brace against the possibility that the lack of more severe changes of the presynaptic organization was due to an inefficient cKO, we measured miniature excitory postsynaptic currents (mEPSCs). We performed voltage clamp recordings in the same primary hippocampal cultures shown before (Figures 1–3A,B), and analyzed 50–100 consecutive mEPSCs in each neuron (Figures 3C,E). We found about 80% longer IEIs in β-Nrxn-deficient (Cre) compared to Cre*^mut^* control neurons (Cre*^mut^*: 46.8 ± 2.1 ms, *n* = 575/7; Cre: 83.7 ± 6.7 ms, *n* = 647 events/7 cells; *p* < 0.001). In contrast, the amplitude and kinetics of the mEPSCs were not affected by the lack of β-Nrxns (Figures 3D,F) because no differences were seen for mean amplitudes (Cre*^mut^*: 45.7 ± 1.1 pA, Cre: 46.9 ± 1.2 pA; *p* = 0.4731), rise-time (Cre*^mut^*: 0.49 ± 0.01 ms, Cre: 0.48 ± 0.01 ms; *p* = 0.57), and half-width (Cre*^mut^*: 1.05 ± 0.03, Cre: 1.11 ± 0.03; *p* = 0.16). Together, the difference in mEPSC frequency without changes in maximal amplitudes and kinetics indicated that our lentivirus-mediated deletion in primary hippocampal cultures was effective, and the degree of deficiency was comparable to the results found in an earlier investigation of other neuronal subpopulations lacking β-Nrxns (Anderson et al., 2015).

**FIGURE 4:**
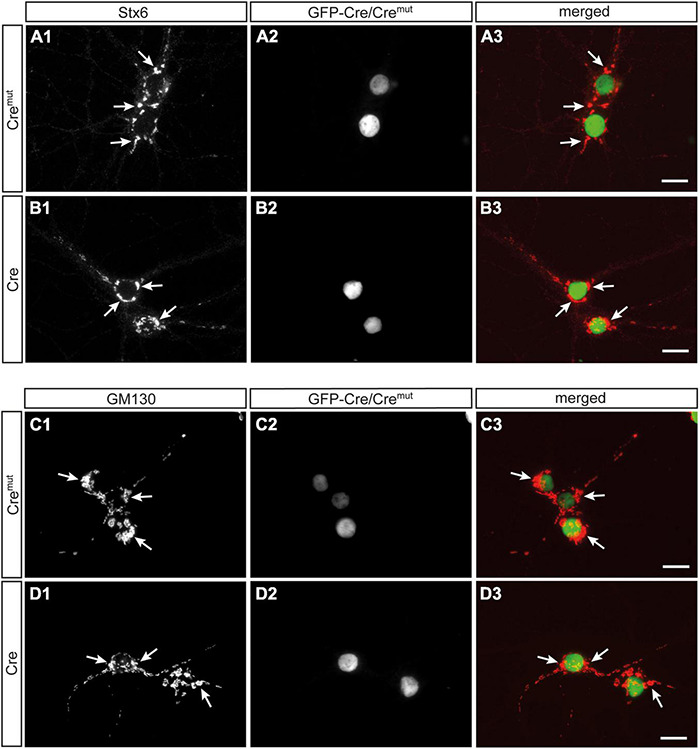
Unchanged Golgi marker expression in β-Nrxn-deficient hippocampal neurons. **(A,B)** Primary hippocampal neurons from floxed β-KI mice were transduced with Cre*^mut^* **(A_1_–A_3_)** or Cre **(B_1_–B_3_)** expressing lentivirus and immunostained at DIV18 with anti-syntaxin 6 (Stx6) as a marker protein of the *trans*-Golgi network (tGN, **A_1_,B_1_**). Successful transduction with lentivirus is demonstrated by nuclear expression of inactive Cre*^mut^* or active Cre recombinase (Cre) fused to GFP **(A_2_,B_2_)**. Merged images **(A_3_,B_3_)** show similar distribution of Stx6 in the Golgi apparatus. Arrows, examples of Stx6-postive clusters. Scale bars: 20 μm. **(C,D)** Cultured neurons as in panels **(A,B)** but immunostained against the *cis*-Golgi marker protein GM130 **(C_1_,D_1_)** with GFP-Cre autofluorescence indicating successful transduction of labeled neurons **(C_2_,D_2_)**. Merged images **(C_3_,D_3_)** reveal no difference in the absence of β-Nrxn. Arrows, examples of GM130-postive clusters. Scale bars: 20 μm.

In contrast to SVs, DCVs are continuously generated and filled with cargo at the Golgi apparatus and then trafficked from the soma to distal release sites (Bharat et al., 2017). Accordingly, reduced numbers of DCVs in presynaptic terminals as observed here (Figure 2) could be due to impaired generation. Such a defect was previously reported in Ca^2+^-dependent activator protein for secretion (CAPS1) KO mice and mechanistically associated with alterations in the *trans*-Golgi network (tGN) (Sadakata et al., 2013). To explore if the phenotype in β-Nrxn-deficient neurons is due to similar changes of the tGN, we compared in β-Nrxn cKO and control cultures the distribution of syntaxin 6 (Stx6) as a tGN marker protein and GM130 as a *cis*-Golgi protein, using the same markers as the earlier study (Sadakata et al., 2013). We observed robust Golgi staining for both markers with a characteristically clustered fluorescence pattern in the soma of Cre*^mut^*-infected control and Cre-infected β-Nrxn cKO neurons with no apparent differences (Figures 4A_**1**_,B_**1**_,C_**1**_,D_**1**_). Green-fluorescent nuclei confirmed proper infection of neurons with lentivirus particles (Figures 4A_**2**_,B_**2**_,C_**2**_,D_**2**_; and merged images, Figures 4A_**3**_,B_**3**_,C_**3**_,D_**3**_). These data indicate that the diminished ChrgA fluorescence and the reduced number of DCVs in β-Nrxn-deficient neurons are likely not due to alterations in the morphology of the Golgi apparatus.

### Reduced Number of Presynaptic Dense-Core Vesicles in Cerebellar Tissue of β-Neurexin Knockout Mice

Our finding of the requirement of β-Nrxns for normal numbers of DCVs in presynaptic boutons was unexpected and striking because these molecules was not previously been linked to DCVs. In contrast, the role of β-Nrxns in synaptic vesicle release and their synaptogenic activity involving transsynaptic interactions has been intensely investigated (Reissner et al., 2013; Sudhof, 2017; Gomez et al., 2021). While cultured neurons have become a widely used tool to monitor DCV distribution and function in KO models (Sadakata et al., 2013; Dominguez et al., 2018; Persoon et al., 2018, 2019), we aimed to validate our DCV phenotype from primary neurons in a more native environment of brain tissue. For this *in vivo* analysis, we selected the highly structured cortex of the cerebellum because it allows the analysis of defined synapse populations (Xiao and Scheiffele, 2018) and it contains high expression of Nrxn-2β and Nrxn-3β in addition to Nrxn-1β, which dominates in the hippocampus (Anderson et al., 2015; Fuccillo et al., 2015; Schreiner et al., 2015). Specifically, we analyzed the DCV distribution in the molecular layer of the cerebellum from adult constitutive β-Nrxnβ-TKO mice in comparison to floxed β-Nrxn knock-in (β-KI) controlsβ-KI. Constitutive β-TKO mice were generated from floxed β-KI animals by breeding with a Cre recombinase deleter strain [B6.FVB-Tg (EIIa-cre) C5379Lmgd/J] to achieve germline deletion of all β-Nrxns prior to this study (Klatt et al., 2021).

**FIGURE 5:**
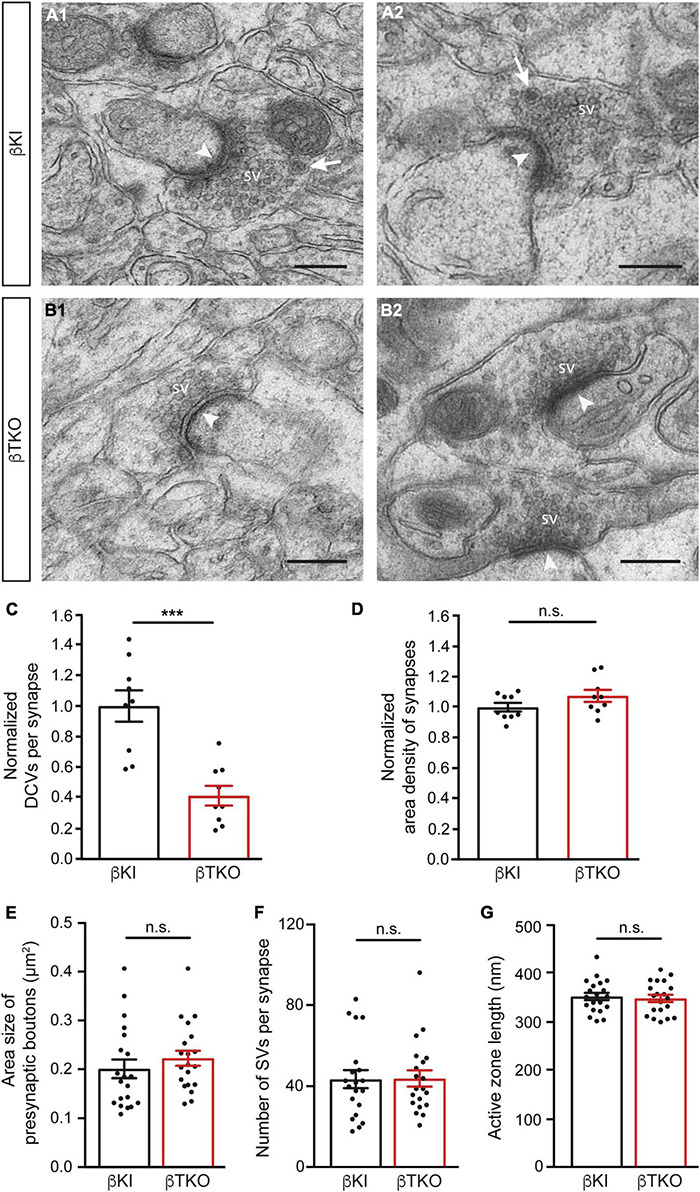
Reduced DCV numbers in cerebellar parallel fiber terminals of β-Nrxn-deficient mice. **(A1,A2)** Representative electron microscopic images of type 1 synapses in the cerebellar molecular layer of β-KI control mice, likely corresponding to excitatory parallel fiber terminals of granule cells. Arrows point to DCVs at the periphery of synaptic vesicle clusters. SV = synaptic vesicle clusters; arrowheads, postsynaptic densities. **(B1,B2)** Similar images as in panel **(A1,A2)** from β-Nrxn-deficient β-TKO cerebellum. Scale bar, for panels **(A,B)**, 250 nm. **(C,D)** Histograms showing the number of DCVs **(C)** and the area density of type 1 excitatory synapses **(D)** in the molecular cell layer from β-KI control (black bars) and β-Nrxn-deficient β-TKO (red bars) cerebella. Data are normalized to control values and shown as mean ± SEM (for actual values, see section “Results”), dots indicate individual data points. Measurements are based on *n* = 9 cerebellar regions from three mice per genotype, corresponding to 3,123 μm^2^ of total area investigated; significance difference indicated as ***p = 0.0002 or n.s. = non-significant, two-tailed unpaired *t*-test. **(E–G)** Histogram summarizing the average area size **(E)**, average number of SVs **(F)**, and average length of the active zone **(G)** of presynaptic boutons from β-KI control (black bars) and β-Nrxn-deficient β-TKO (red bars) parallel fiber terminals. Samples as in panels **(C,D)**, data are shown as mean ± SEM; significance difference indicated as n.s. = non-significant, two-tailed unpaired *t*-test.

In the mature cerebellum, the molecular layer predominantly contains two types of excitatory synapses, the majority originating from granule cells *via* parallel fibers and a minority from climbing fibers of the inferior olive neurons (Napper and Harvey, 1988; Xu-Friedman et al., 2001). We studied the ultrastructure of boutons terminating on postsynaptic spines of the proximal part of dendrites arising from β-KI (Figures 5A_**1**_,A_**2**_) and β-TKO (Figures 5B_**1**_,B_**2**_) Purkinje cells and observed a similar population of boutons with clustered SVs, postsynaptic density (arrowheads), frequent mitochondria, and engulfing astrocytic processes. In β-KI control boutons, usually a single DCV per presynaptic terminal was found at the periphery of SVs (arrows, Figures 5A_**1**_,A_**2**_). However, in β-Nrxn-deficient β-TKO cerebella, most boutons seemed to be devoid of DCVs (“empty synapses,” Figures 5B_**1**_,B_**2**_), corresponding to the shift from boutons with 1 or 2 DCVs to those without any DCV observed in cultured hippocampal neurons (Figure 2). Quantification confirmed this impression and revealed a 50% reduction of DCV numbers in presynapses (Figure 5C; β-KI control: 0.25 ± 0.03 DCVs/presynapse; β-TKO: 0.1 ± 0.02, *p* = 0.0002). As the area density of excitatory synapses, i.e., the number of synapses per area, was unchanged (Figure 5D; β-KI control: 77.8 ± 2.2 synapses/225 μm^2^; β-TKO: 83.6 ± 3.0, *p* = 0.17), our results indicate a specific reduction of the number of DCVs in the intact cerebellum, consistent with the quantitative data from primary hippocampal neurons (Figure 2D).

The release of neuropeptides such as BDNF or neuropeptide Y has not only been linked to the modulation of synaptic transmission but also to changes in synaptic plasticity and ultrastructure (McAllister et al., 1999; Poo, 2001; van den Pol, 2012). As most synapses analyzed here likely correspond to parallel fiber terminals (Napper and Harvey, 1988; Xu-Friedman et al., 2001), we tested whether the reduction of DCVs in β-Nrxn-deficient cerebellar granule cells (CGCs) has an effect on their synapse structure by measuring important parameters of their ultrastructure. However, no differences between constitutive β-Nrxn-deficient and β-KI control boutons could be determined for presynaptic area size (Figure 5E; β-KI control: 0.2 ± 0.02 μm^2^, β-TKO: 0.22 ± 0.12, *p* = 0.3808), number of synaptic vesicle (Figure 5F; β-KI control: 43.7 ± 4.37 vesicles/synapse, β-TKO: 44.0 ± 3.96, *p* = 0.9530) or active zone length (Figure 5G; β-KI control: 351.2 ± 7.5 nm, β-TKO: 351.2 ± 7.5, *p* = 0.9530). These results suggest that the reduction of the number of presynaptic DCVs in β-Nrxn-deficient mice did not impair the general differentiation or maintenance of terminals, in line with earlier analyses of this mouse model (Anderson et al., 2015; Klatt et al., 2021).

**FIGURE 6:**
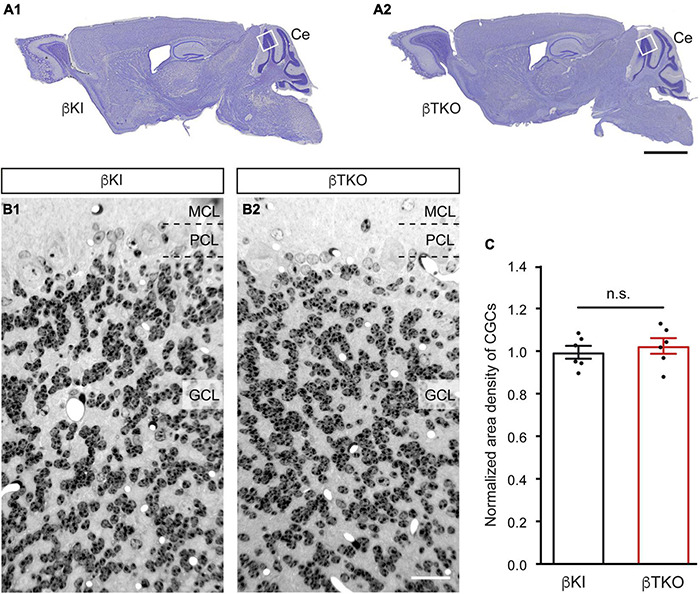
Normal numbers of cerebellar granule cells (CGCs) in β-Nrxn-deficient mice. **(A)** Nissl-stained parasagittal sections through the brains of β-KI control **(A_1_)** and β-Nrxn-deficient β-TKO **(A_2_)** mice. Ce = cerebellum; box indicates approximate position of area investigated in panels **(B,C)**. Scale bar: 2 mm. **(B)** Representative images of 1-μm semithin sections from β-KI control **(B_1_)** and β-Nrxn-deficient β-TKO **(B_2_)** cerebellum stained with toluidine blue dye. GCL, granule cell layer; PCL, Purkinje cell layer; MCL, molecular cell layer. Scale bar: 125 μm. **(C)** Histogram showing the area density of cells in the GCL of the cerebellum of β-KI control (black bars) and β-Nrxn-deficient β-TKO (red bars) mice. Number of granule cells was counted in a 1 mm^2^ area and β-TKO data normalized to control. Data are shown as mean ± SEM, dots indicate individual data points, measurements are based on *n* = 6 areas from three animals per genotype; significance difference indicated as n.s. = non-significant, two-tailed unpaired *t*-test.

**FIGURE 7:**
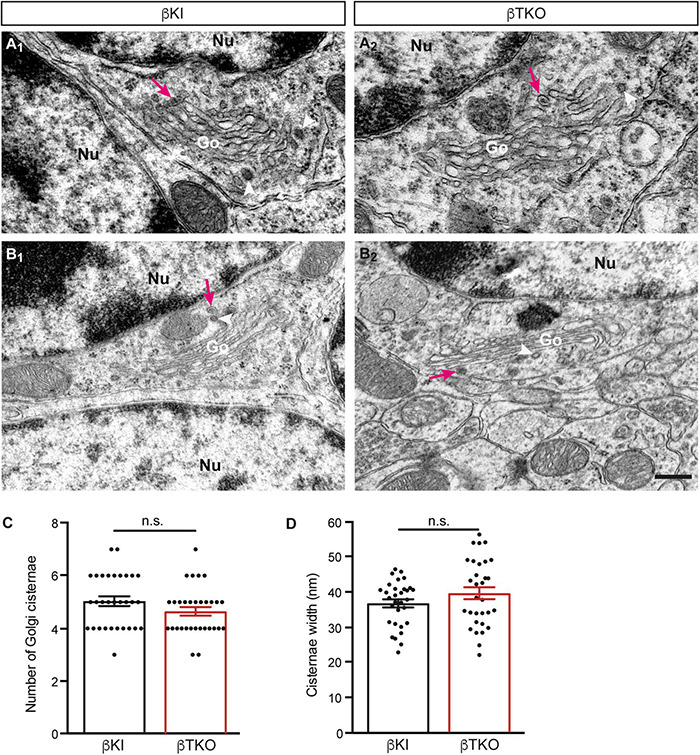
Normal Golgi morphology in CGCs of β-Nrxn-deficient mice. **(A_1_,A_2_)** Representative electron microscopic images of the Golgi apparatus (Go) of CGCs from β-KI control mice. Note visible DCVs (magenta arrows) in the vicinity of Golgi stacks. Nu, nucleus; arrowheads, coated vesicles. **(B_1_,B_2_)** Similar images as in panels **(A_1_,A_2_)** but from β-Nrxn-deficient β-TKO cerebellum. Labels as in panels **(A_1_,A_2_)**. Scale bar for all images: 250 nm. **(C,D)** Histograms showing the number of cisternae **(C)** and the width of cisternae **(D)** on random cross-sections of the GCL from β-KI control (black bars) and β-Nrxn-deficient β-TKO (red bars) cerebella. Data are shown as mean ± SEM, dots indicate individual data points. Measurements are based on *n* = 31 Golgi apparatus from three mice per genotype; significance difference indicated as n.s. = non-significant, two-tailed unpaired *t*-test.

To finally brace against the possibility that the reduction of DCVs in the β-TKO are due to cell death of entire CGCs or alterations of their Golgi apparatus, required for the generation of DCVs (Bharat et al., 2017), we studied the granule cell layer (GCL) at the light-microscopic level and the integrity of the Golgi apparatus at the ultrastructural level. The cerebella of β-KI control and β-TKO mice were gross anatomically normal, similar to the rest of the overall brain structures, as judged from the comparison of β-KI control and β-TKO mice (Figures 6A_**1**_,A_**2**_). To exclude more subtle changes of the neuronal population relevant to the phenotype of reduced DCVs in parallel fiber boutons, we studied cell numbers in semithin sections of the GCL from β-KI control and β-TKO cerebella (Figures 6B_**1**_,B_**2**_). Our quantification revealed no difference in the area density of CGCs (Figure 6C, data normalized to β-KI control: 1.0 ± 0.03 cells/mm^2^, β-TKO: 1.03 ± 0.04, *p* = 0.5490). To exclude ultrastructural alterations of the Golgi apparatus, we finally compared critical parameters of Golgi morphology on random cross-sections of CGCs from β-KI controls (Figures 7A_**1**_,A_**2**_) to β-TKO neurons (Figures 7B_**1**_,B_**2**_) by electron microscopy. No major differences of the extent or organization of *cis*- or *trans*-Golgi regions or dilatation of cisternae were observed, suggesting an intact morphology of the Golgi apparatus in the absence of β-Nrxns. Specifically, we found an unchanged number of Golgi cisternae (Figure 7C; β-KI control: 5.03 ± 0.18, β-TKO: 4.65 ± 0.16, *p* = 0.1201) and similar cisternae width (Figure 7D; β-KI control: 36.72 ± 1.15 nm, β-TKO: 39.6 ± 1.71, *p* = 0.1673). As expected, these dimensions are consistent with the earlier reports that determined, for example, an average width of cisternae of about 38 nm (Emperador-Melero et al., 2018). Moreover, DCVs with a diameter between 68 and 79 nm, i.e., within the determined range for presynaptic DCVs as shown above, could be identified in the vicinity of Golgi apparatus in both β-KI controls (arrows, Figures 7A_**1**_,A_**2**_) and β-TKO neurons (arrows, Figures 7B_**1**_,B_**2**_). However, it was not possible to reliably quantify the number of DCVs after generation at the tGN by electron microscopy because the probability of finding DCVs next to a Golgi apparatus on cross-sections was extremely low, limiting the sample size needed for a thorough investigation. Thus, the analysis of the maturation and trafficking of DCVs in β-Nrxn-deficient neurons will have to be done by entirely different methods. In conclusion, our results from the cerebellar tissue confirm the data from cultured hippocampal neurons, and, together, unequivocally demonstrate that the deletion of β-Nrxns specifically alters the distribution of presynaptic DCVs in neurons.

## Discussion

### Reliability, Validity, and Plausibility of the Dense-Core Vesicle Phenotype in β-Neurexin-Deficient Neurons

This study is the first that links a member of the most widely investigated synaptic cell adhesion molecules (β-Nrxn) to DCVs in neurons. We mostly focused our analysis on DCVs in presynaptic boutons of excitatory neurons because (i) β-Nrxns have a known function in the differentiation of these presynaptic terminals and their Ca^2+^-dependent release (Dean et al., 2003; Anderson et al., 2015; Born et al., 2015; Klatt et al., 2021) and (ii) DCVs are preferentially located and released at high rates at synapses compared to extrasynaptic sites or dendrites in hippocampal neurons (van de Bospoort et al., 2012; Tao et al., 2018). Our main conclusion is supported by several lines of evidence that the normal distribution of DCVs in presynaptic boutons depends on β-Nrxns.

First, the numbers of presynaptic DCVs upon the deletion of β-Nrxns are clearly reduced by about 50% from control values. These control values (wild type or Cre*^mut^*) are similar to other studies. The majority, or 60%, of wild-type hippocampal synapses have no/zero DCVs (Dominguez et al., 2018), remarkably similar to the 59% of synapses without DCV determined in our study (Figure 2D). Only a minority of terminals contain one DCV, for example, 25% of synapses as determined by Dominguez et al. (2018), or 24% in our study (Figure 2D); and only a few boutons have two or more DCVs. These numbers are also consistent with additional studies that reported between 45 and 70% of synapses without any DCV (van de Bospoort et al., 2012; Persoon et al., 2018). Surprisingly, a large fraction of wild-type boutons without DCVs might indicate that the capacity of releasing neuromodulators is strictly controlled in hippocampal neurons. Based on this consideration, the phenotype we observed in β-Nrxn KO, i.e., shifting a subpopulation of boutons from 1 to 0 DCV (Figure 2D), might represent a bold change in the function of the neuronal network.

Second, the phenotype of reduced numbers of presynaptic DCVs upon the deletion of β-Nrxns is specific as it can be found in two different neuronal populations that normally contain high levels of β-Nrxn, excitatory hippocampal (Figures 1, 2) and cerebellar (Figure 5) neurons, and their synaptic boutons. Neurons from these two different brain regions were chosen as Nrxn-1β is the prominent isoform in the hippocampus with lower levels of Nrxn-2β and Nrxn-3β, whereas in the cerebellum, there were higher levels of Nrxn-2β and Nrxn-3β in addition to Nrxn-1β (Anderson et al., 2015; Fuccillo et al., 2015; Schreiner et al., 2015). The phenotype also appears specific because the deletion of β-Nrxns neither affected the distribution of a presynaptic marker protein, Bassoon (Figure 3), nor the number of synapses (Figure 5D) or ultrastructural parameters such as presynaptic bouton size (Figure 5E), number of SVs (Figure 5F), and the length of active zone (Figure 5G), confirming earlier investigations of β-Nrxn-deficient neurons (Anderson et al., 2015; Klatt et al., 2021).

Third, the phenotype is robust because it can be observed by independent experimental methods. Reduced levels of ChrgA, an intravesicular matrix protein of DCVs (Bharat et al., 2017; Dominguez et al., 2018), were shown by immunofluorescence intensity measurements (Figure 1), and the reduced numbers of ultrastructurally identified presynaptic DCVs were demonstrated using electron microscopy (Figures 2, 5). In fact, the 50% reduction seen here upon the deletion of β-Nrxns is higher than the 30% reduction of DCVs reported in an investigation of CAPS1 KO, which established a new function for CAPS1 (Sadakata et al., 2013). ChrgA and its closely related isoform Chromogranin B, in turn, have been widely used as marker proteins of DCVs in brain neurons (Machado et al., 2010; Bartolomucci et al., 2011). Interestingly, the deletion of these molecules did not compromise the ability of DCVs to fuse with the plasma membrane upon bursts of action potentials (Dominguez et al., 2018). Moreover, the molecules are not essential for DCV distribution to synapses because the deletion of both ChrgA and B in mice revealed a normal number of synaptic DCVs (Dominguez et al., 2018). These results indicate that the reduction of DCVs reported in our study is a direct consequence of deleting β-Nrxns and not a secondary effect of downregulating the expression or loading of DCVs with ChrgA.

Fourth, the phenotype is reliable because it was present in similar magnitude in cultured primary neurons conditionally deleted for β-Nrxns by Cre recombinase-expressing lentivirus (Figure 2) and in the brain tissue from constitutive β-TKO β-TKO (Figure 5). Thus, DCV numbers in presynaptic boutons drop by 50% when β-Nrxns are missing, as shown by quantitative electron microscopy. This is a surprising finding because previous studies have solely identified a role of β-Nrxns in the release of small SVs in cKO neurons, including reduced presynaptic Ca^2+^ influx and glutamate release, diminished spontaneous mini frequencies, and impaired endocannabinoid signaling (Anderson et al., 2015; Klatt et al., 2021). The clear effect of β-Nrxns on DCV distribution has thus far escaped attention but is a remarkable finding due to the low amount of β-Nrxn protein in the brain, estimated for Nrxn1β at ≈0.9 fmol/μg compared to N-cadherin (≈20 fmol/μg) or PSD-95 (≈54 fmol/μg) (Schreiner et al., 2015). The low abundance translates into very low copy numbers at individual terminals, estimated at 7–16 molecules per synapse for all β-Nrxn isoforms combined. Together with our recent observation that a large extrasynaptic axonal pool of these elusive molecules exists (Klatt et al., 2021), the numbers imply that very few copies of a β-Nrxn variant are present at individual synapses at a given time point. However, β-Nrxns are also highly mobile molecules and only transiently confined at boutons (Klatt et al., 2021), opening the possibility that the dynamic behavior of synaptic and axonal β-Nrxns is involved in the regulation of DCVs.

### Regulation of Dense-Core Vesicles by the Presynaptic Organizer Molecules β-Neurexins

We did not obtain any evidence that ChrgA immunofluorescence clusters were differently distributed in β-Nrxn-deficient primary hippocampal neurons compared to controls (Figures 1, 3). As the pattern of fluorescence was identical in β-Nrxn-deficient (Cre) vs. Control (Cre*^mut^*) neurons (Figures 1D_**1**_,D_**2**_), an ectopic accumulation seems unlikely. In support, we also did not see the accumulation of DCVs in axons outside synapses by EM (Figure 2). In fact, DCVs are very sparsely distributed in “inter-bouton parts of axons” both in control and β-Nrxn-deficient neurons as seen in our EM samples (Figure 2) and reflected by the lower ChrgA fluorescence intensity (Figure 1). Thus, the deletion of β-Nrxns and the concomitant reduction of spontaneous and evoked synaptic release as shown here (Figures 3C–F) and elsewhere (Anderson et al., 2015; Klatt et al., 2021) leads to a reduction of presynaptic DCVs without ectopic accumulation. This is a different scenario, for example, compared to chronic inactivity by the application of TTX, which caused the accumulation of presynaptic DCVs in a recent study of cultured hippocampal neurons by cryo-EM tomography (Tao et al., 2018).

We also did not observe an accumulation of DCVs around the Golgi apparatus in β-TKO neurons where these organelles are loaded with cargo and budded from tGN cisternae (Figure 7). The unchanged localization of Golgi markers (Figure 4), identical cell density (Figure 6), and intact morphology of the Golgi apparatus (Figure 7) rather suggest that biogenesis of DCVs in general does not depend on β-Nrxns. For quantification of putative changes of the Golgi ultrastructure, we measured the number of Golgi cisternae and diameter of cisternae width (Figures 7C,D), using the same parameters that led to the discovery of a trafficking phenotype of DCVs in Vti1-deficient neurons (Emperador-Melero et al., 2018). The undisturbed Golgi organization accompanying the phenotype of reduced numbers of presynaptic DCVs in the absence of β-Nrxns is striking because the only other study reporting a comparable defect of DCV numbers actually depended on altered Golgi structure and impaired trafficking (Sadakata et al., 2013). In this study, the analysis of cKO mice of the CAPS1 revealed a 30% reduced immunoreactivity for the DCV marker secretogranin II, another member of the granin protein family (Bartolomucci et al., 2011), and a 50 or 70% reduction of the number of presynaptic DCVs in the cerebellum or hippocampus, respectively (Sadakata et al., 2013). While these data are similar to our findings here, CAPS1-deficient mice additionally showed altered expression of the tGN marker protein syntaxin6, dilated tGN cisternae, and reduced numbers of SVs (Sadakata et al., 2013), all of which were absent in our β-Nrxn-deficient neurons. Moreover, other studies of CAPS1-deficient mammalian chromaffin cells and neurons have pointed to an essential role for survival and concluded that it functions strongly in the priming or fusion of DCVs (Liu et al., 2010; Farina et al., 2015; Crummy et al., 2019). In any case, a comparison of these partially overlapping, partially distinct phenotypes indicates that CAPS1 primarily plays a role in the generation, trafficking, and fusion of DCVs, whereas β-Nrxns appear to have a more distinct, regulatory role.

It has been shown that DCVs are highly mobile organelles that undergo long-range translocation and can be captured at the level of individual boutons (Wong et al., 2012), a process that involves phosphorylation of synaptotagmin-4 at presynapses (Bharat et al., 2017) and liprin-α at postsynaptic spines (Stucchi et al., 2018). In fact, the capture of DCVs at active terminals is an important process that might complement the statistically significant presynaptic enrichment of these organelles as shown by Poisson statistics based on randomness (Robinson et al., 2016). Relevant to a presynaptic phenotype, it was demonstrated that the capture of DCVs in hippocampal neurons requires destabilization of DCV/Syt4/KIF1a complexes and increased neuronal activity (Bharat et al., 2017). β-Nrxns themselves depend on microtubule KIF1a-dependent trafficking (Neupert et al., 2015), and synaptic release is reduced in β-Nrxn-deficient neurons as shown here (Figures 3C–F) and earlier (Anderson et al., 2015; Klatt et al., 2021). Consequently, the inability to redirect DCVs from the circular trafficking pool to active boutons (Wong et al., 2012; Bharat et al., 2017) could explain the observations described in our study. Alternatively, the reduced number of DCVs could imply that less active zone material is delivered to active synapses (Tao et al., 2018), reducing the release probability and the readily releasable pool of vesicles, possibly reflected by the reduced mEPSC frequency or elevated IEI (Figure 3E). However, independent of the mechanism that causes the reduction of presynaptic DCVs in β-Nrxn-deficient neurons, it can be predicted that shifting a population of synapses from 1 to 0 DCV (Figure 2D) means a reduction of the neuromodulatory prowess, which in turn might represent a bold change in the function of the neuronal network. Thus, future studies will have to dissect the functional role of β-Nrxns for neuropeptide or neurohormone release.

## Data Availability Statement

The original contributions presented in the study are included in the article/supplementary material, further inquiries can be directed to the corresponding authors.

## Ethics Statement

The animal study was reviewed and approved by Landesamt für Natur, Umwelt und Verbraucherschutz (LANUV, NRW, Germany).

## Author Contributions

AR and MM: conceptualization, methodology, and project administration. SF, JB, and AR: investigation and formal analysis. AR, SF, JB, and MM: writing-original draft, review, and editing. MM: funding acquisition. All authors approved the submitted version.

## Conflict of Interest

The authors declare that the research was conducted in the absence of any commercial or financial relationships that could be construed as a potential conflict of interest.

## Publisher’s Note

All claims expressed in this article are solely those of the authors and do not necessarily represent those of their affiliated organizations, or those of the publisher, the editors and the reviewers. Any product that may be evaluated in this article, or claim that may be made by its manufacturer, is not guaranteed or endorsed by the publisher.
